# Supplementary material for: Development of BCMA-Targeted Bispecific Natural Killer Cell Engagers for Multiple Myeloma Treatment
Source: Antibodies (Basel). 2024 Nov 29;13(4):97. doi: 10.3390/antib13040097 (PMC11672634; doi:10.3390/antib13040097)
Supplement: Supplementary file 1 [file antibodies-13-00097-s001.zip › antibodies-3266938-supplementary.pdf]

Supplementary Materials for

Development of BCMA-Targeted Bispecific Natural Killer Cell Engagers for Multiple Myeloma Treatment

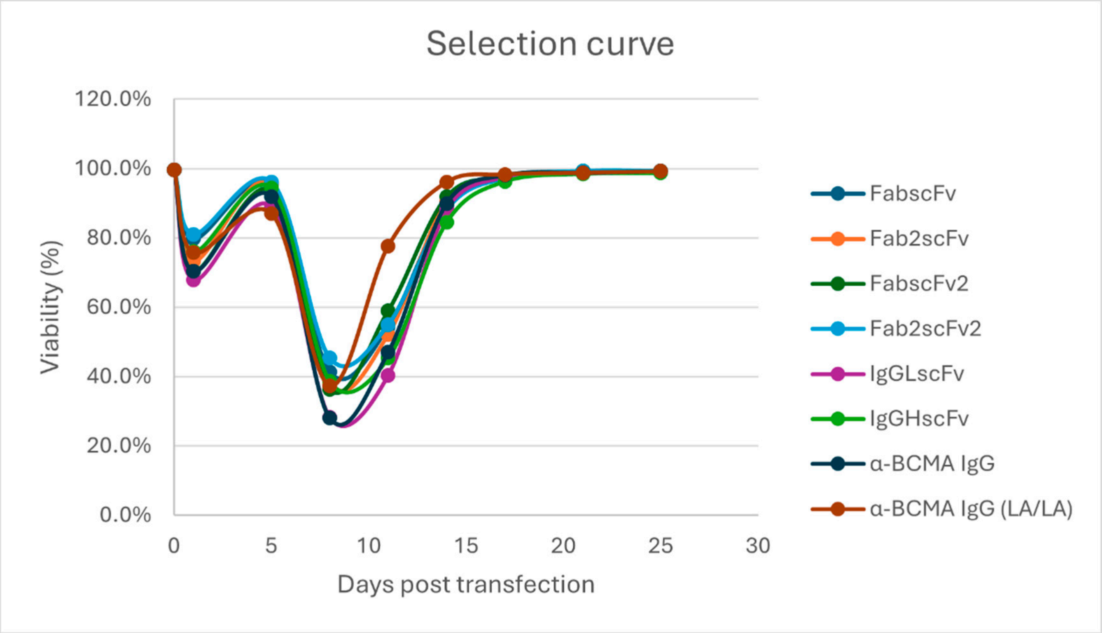

Supplementary Figure S1: Cytotoxicity curve of puromycin treatment for the 8 CHO cell pools.

Calibration Details	IgG			
Calibration Type	Lin, WithOffset		*Offset (C0)*	-1.7896
Evaluation Type	Area		*Slope (C1)*	173.4429
Number of Calibration Points	37		*Curve (C2)*	0.0000
Number of disabled Calibration Points	0		*R-Square*	0.9991

Calibration Plot	IgG

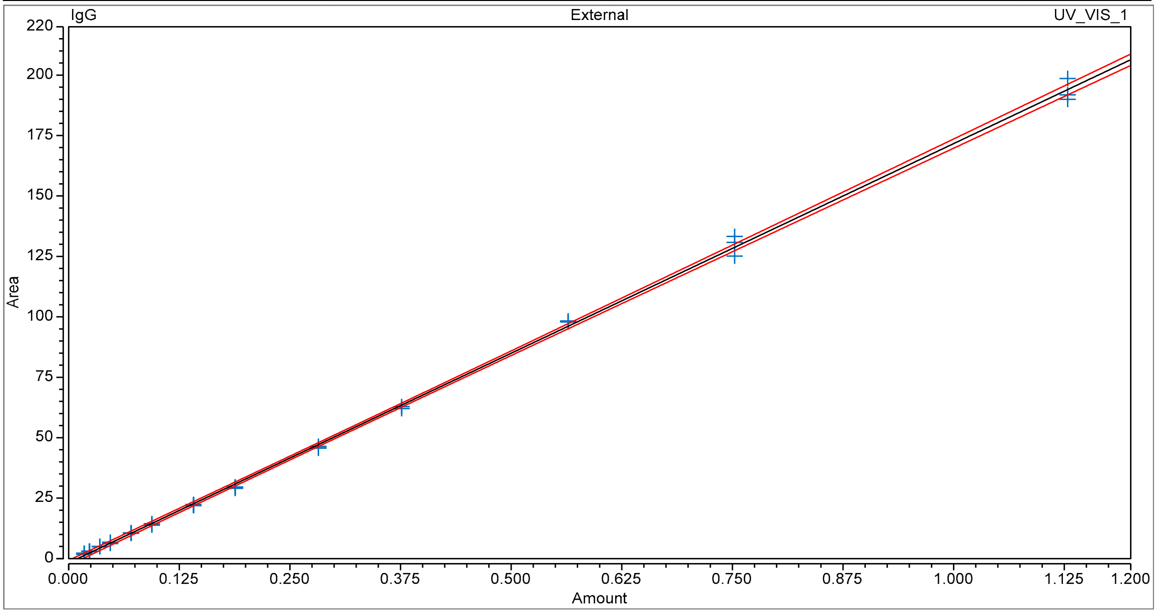

Supplementary Figure S2: Calibration curve of HPLC-SEC.
